# Patients’ preferences for involvement in the decision-making process for treating diabetic retinopathy

**DOI:** 10.1186/s12886-017-0526-z

**Published:** 2017-08-09

**Authors:** Lydia Marahrens, Raimar Kern, Tjalf Ziemssen, Andreas Fritsche, Peter Martus, Focke Ziemssen, Daniel Roeck

**Affiliations:** 1 Center for Ophthalmology, Eberhard Karls University of Tuebingen, Tuebingen, Germany; 2 Department of Neurology, Center of Clinical Neuroscience, University Hospital Carl Gustav Carus, Dresden University of Technology, Dresden, Germany; 3 German Centre for Diabetes Research (DZD), Institute for Diabetes Research and Metabolic Diseases of the Helmholtz Centre Munich at the University of Tuebingen, Tuebingen, Germany; 4 Institute of Clinical Epidemiology and applied Biostatistics University of Tuebingen, Tuebingen, Germany

**Keywords:** Adherence, Diabetic retinopathy, Decision-making, Treatment, Disease knowledge

## Abstract

**Background:**

To assess factors associated with the preferred role of the attending ophthalmologist in the decision-making processes before treating diabetic retinopathy (DR).

**Methods:**

Cross-sectional study of 810 adults attending secondary diabetes care centers (NCT02311504). Diabetes patients were classified using a validated questionnaire in an ophthalmologist-dominant decision-making (ODM), shared decision-making (SDM) and patient-dominant decision-making (PDM) style. Multivariate logistic regression was performed to determine factors associated with the decision-making process.

**Results:**

A majority of 74.3% patients preferred SDM between ophthalmologist and patient, 17.4% patients wanted ODM, delegating the decision-making process to the ophthalmologist, 8.3% preferred the autonomous style of PDM. Patients wanting ODM were older (OR = 1.2 per decade, *p* = 0.013), had a lower level of education (OR = 1.4, *p* = 0.001) and had a higher frequency of consultations per year (OR = 1.3, *p* = 0.022). Patients with better basic knowledge in DR and memorizing their HbA_1_c level showed a higher propensity for SDM (OR = 1.1, *p* = 0.037).

Patients wanting PDM had a significantly higher education (OR = 1.3, *p* = 0.036) and a greater desire for receiving information from self-help groups (OR = 1.3, *p* = 0.015).

**Conclusions:**

The first evaluation of the general patient wishes for the treatment of DR confirmed the concept of SDM, which was favored by three quarters. In particular, older patients with low educational attainment wanted to delegate the decision-making process to the ophthalmologist. Amelioration of ophthalmologic education in diabetic programs might take up patients’ propensity for SDM. Regardless of the decision-making group, nearly all patients wanted the medical and scientific information to be transferred by and shared with the ophthalmologist.

**Trial registration:**

The study was registered on www.clinicaltrials.gov (identifier: NCT02311504) on December 4th 2014.

**Electronic supplementary material:**

The online version of this article (doi:10.1186/s12886-017-0526-z) contains supplementary material, which is available to authorized users.

## Background

The patient with a loss of vision caused by diabetic macular edema has to face several questions: *With regard to the individual’s situation, are the available treatments and drugs equally effective?* [1] *Do the lower risks of cataract formation and glaucoma with anti-VEGF drugs counterbalance the potentially lower frequency (treatment burden), reported for intravitreal steroids? What is the role of the focal laser?* [2, 3] *What is the best systemic treatment?* [4].

Formerly, medical decisions were made on behalf of patients by the ophthalmologist after careful deliberation in a paternalistic way, resulting in ‘immature’ and passive patients [5, 6]. However, although medical emergencies and acute diseases like bacterial infections or chemical burns normally still require an immediate and paternalistic decision on the part of the ophthalmologist [7, 8], such behavior minimizes the individual’s responsibility and their adherence in chronic diseases [9, 10]. In acute illnesses, but even more in chronic diseases, the active and informed involvement of patients in managing their diseases is becoming increasingly important, and is today, especially in diabetes, the standard for good medical care [7, 11–14]. The trend towards more individual autonomy, self-determination and personal responsibility as promoted by the *“Choosing Wisely”* initiative is an ongoing process in our society [15].

Although patient communication and satisfaction have become a stronger focus of attention in ophthalmology during the last decade [9, 16], there is still a huge gap in knowledge about what patients expect and which factors are relevant to the doctor-patient-relationship [17, 18]. There is no study addressing the questions what patients with diabetes expect or wish from the ophthalmologist. Furthermore, there is little knowledge, which sources of information are of importance in this context.

## Methods

We conducted a cross-sectional study of 810 individuals attending secondary diabetes care centers between January 1 and May 1, 2014. Human subject approval was obtained from the institutional review board at the affiliated academic institution. The DiabCheck^OCTplus^ study adhered to the tenets of the Declaration of Helsinki. We certify that all applicable institutional and governmental regulations concerning the ethical use of human volunteers were followed during this research (NCT02311504).

Eligibility criteria included age of more than 18 years and the proven medical diagnosis of diabetes (medical record). Individuals were excluded if mental disability, dementia and poor German language skills were noted.

### Recruitment

Upon arrival for their diabetologist appointment, eligible subjects were invited for a private oral interview by one of two trained study investigators. Of 843 diabetes patients, 831 individuals were eligible for the study, 21 declined to participate because of time constraints or for personal reasons. Therefore, 810 patients agreed to participate in the study. To improve representativeness the first center was located in a high densely populated area of a large metropolitan city, the second in an intermediate densely populated area on its boundary and the third in a thinly populated rural area further away [19]. In the case of visual impairment or reading disabilities, a relative or another person of trust had to be present, when the informed consent form was orally administered and signed. Patients did not receive financial compensation but fundus imaging for their voluntary participation in the study.

### Measures

First, data was collected from patient interviews by means of a questionnaire on sociodemographic characteristics, previous information sources, frequency of ophthalmologist consultations as well as disease knowledge and perceptions. Reliability and validity of the questionnaire were tested in a pilot cohort of 97 subjects prior to conducting the study.

### Sociodemographic and medical characteristics

All three secondary diabetes care centers had implemented a medical information system. Detailed data about patient’s health status, medical condition, diagnosis, treatment and information on their diabetes such as type, duration and laboratory test results were extracted from the patient’s electronic medical record.

To facilitate comparisons, the German education levels were classified and adapted according to the International Standard Classification of Education (ISCED) as follows: a) lower secondary education for up to nine school years, b) intermediate secondary education was declared as a new group because formerly the school system in Germany had an own school-leaving qualification for school graduates after 10 years of education, c) upper secondary education for 11 to 13 school years (ISCED-level 3 and 4) and d) tertiary education with a cumulative period of education of more than 13 years equivalent to bachelor’s, master’s or doctoral level (ISCED-level 5–8) [20].

### Disease knowledge and information sources

To obtain objective information on the level of knowledge about DR and its ocular complications, a score with 10 different items (0–10) was created: five basic and four expanded knowledge questions, and one question about the patients’ knowledge of their HbA_1_c level by heart, all verified for accuracy. Additionally, a patient’s subjective self-assessment about this knowledge was determined using a 5-point Likert scale ranging from one (“not informed at all”) to five (“very good informed”).

To assess patients’ information sources (listed in Fig. 2) and their importance for weighing up pros and cons before treatment of DR a 5-point Likert scale was created ranging from one (“unimportant”) to five (“very important”).

### Operational definition of the decision-making models

**Fig. 1:**
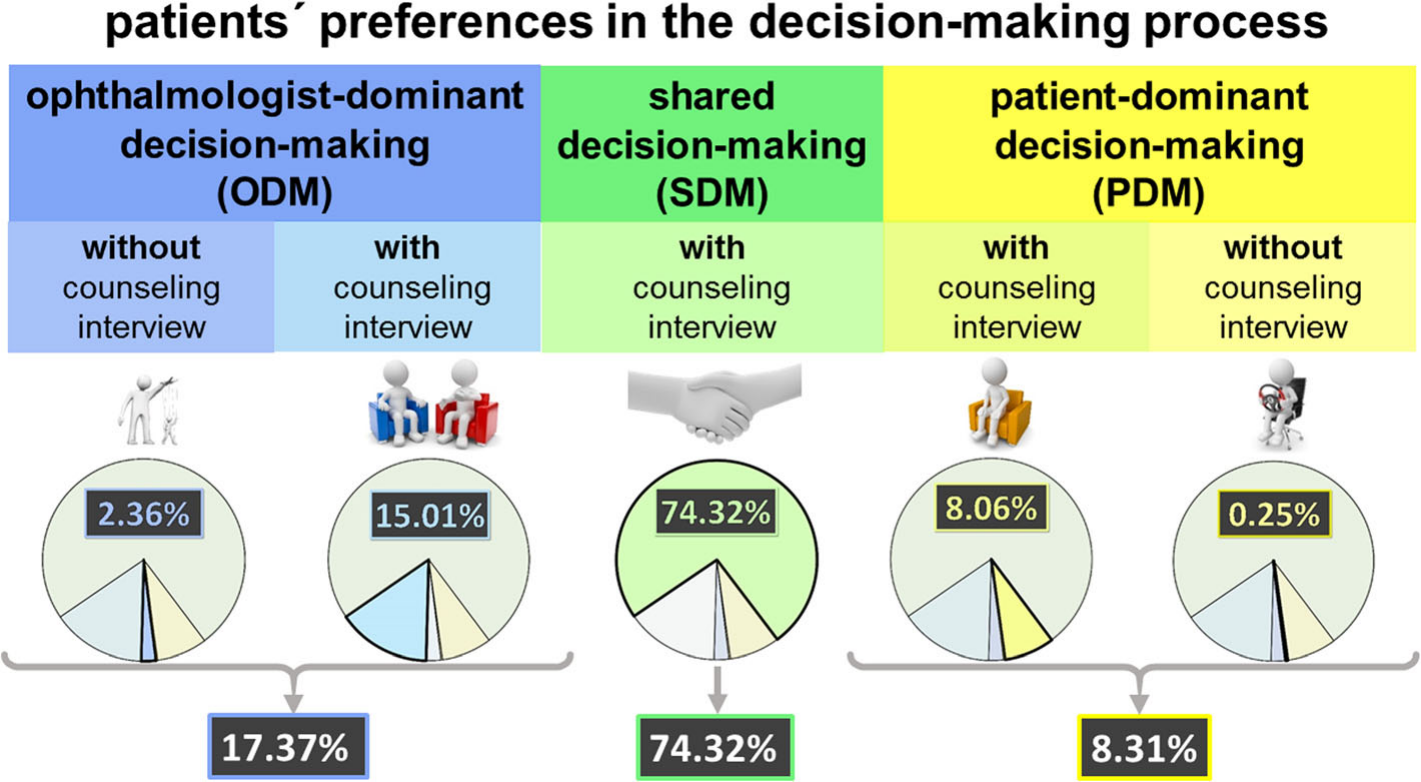
Patients’ preferences in the decision-making process between patient and ophthalmologist, modeled according to Degner and Sloan and Charles et al. [20, 21]

Finally, a hypothetical scenario was described for the subjects by an intelligible-to all introduction (two sentences) that the ophthalmologist recently diagnosed active DR and a therapy decision had to be made. Counseling interviews included detailed treatment information. Patients could choose between five options (Fig. 1) [21]. Following Charles and colleagues, three categories were analyzed in the decision-making process [22]. First was the ophthalmologist-dominant decision-making (ODM) group, whereby the ophthalmologist was the sole deciding authority and the patient assumed a passive role in decision-making, known as the paternalistic model. Second, the shared decision-making (SDM) group, whereby the ophthalmologist and the patient were both actively involved, defined the shared model. Third, the patient-dominant decision-making (PDM) group, whereby the patient played an active role in decision-making and solely determined the preferred treatment, was the informed model.

### Statistical analyses

For categorical outcomes, the χ^2^-test was used to test for significant differences in patients’ characteristics by group. For continuous outcomes, significant differences were evaluated with the non-parametric Kruskal-Wallis test or the one-way ANOVA for normally distributed data. Multivariate logistic regression analysis was used to determine independent parameters in the decision-making process. In all statistical analyses a *p*-value <0.05 was defined as significant. The statistical analyses were performed with SPSS 22 (*IBM Corp. Released 2013. IBM SPSS Statistics for Windows, Version 22.0. Armonk, NY: IBM Corp.*).

## Results

Of the 806 eligible subjects, a majority of 74.3% patients (599) opted for SDM between ophthalmologist and patient with a two-way information exchange. However, 17.4% (140) selected a paternalistic approach with decision-making only by the treating ophthalmologist (Fig. 1).

The preference for PDM was expressed by 8.3% of patients (67). Even those in both groups, aiming for a unilateral decision (23.1%, 186), mostly asked for a counseling interview; 82.6% of patients wanted to have an active role in treatment decision-making, either sharing responsibility for decision-making with the ophthalmologist or being the patient-dominant decision-maker; 97.4% of patients wanted medical and scientific information from the ophthalmologist before treatment of the retina (Fig. 1).

The mean age of the ODM group was 64 years, of the SDM group 58 years, and of the PDM group 57 years; p = <0.001 (Table 1). With increasing age, the patients favored a decision dominated by the ophthalmologist (Additional file 1: Figure S1).

**Table 1:** Univariate analysis of patient characteristics associated with the decision-making groups ODM, SDM and PDM (Totals and subtotals may vary slightly due to missing data)

Characteristics	ODM *N* = 140	SDM *N* = 599	PDM *N* = 67	*p*-value (univariate)
Age - yr. [min-max]	63.94 ± 13.61 [18–90]	57.51 ± 15.53 [18–88]	57.18 ± 15.61 [22–88]	<.001
Gender - no. (%)				
Males	81 (57.9)	327 (54.6)	35 (52.2)	.718
Females	59 (42.1)	269 (44.9)	32 (47.8)	
Diabetes duration - yr	15.99 ± 11.72	15.65 ± 12.03	17.33 ± 13.84	.555
Diabetes type - no. (%)				
Type 1	25 (17.9)	203 (33.9)	24 (35.8)	<.001
Type 2	110 (78.6)	355 (59.3)	41 (61.2)	
HbA_1_c level (mean, %)	7.39 ± 1.14	7.23 ± 1.01	7.36 ± 0.86	.211
Patients knowledge of HbA_1_c no. (%)				
known	97 (69.3)	500 (83.5)	55 (82.1)	.001
not known	38 (27.1)	88 (14.7)	11 (16.4)	
Complications and comorbidities no. (%)				
Hypertension	95 (67.9)	341 (56.9)	38 (56.7)	.069
Dyslipidemia	74 (52.9)	251 (41.9)	24 (35.8)	.535
Neuropathy	63 (45.0)	191 (31.9)	16 (23.9)	.003
Retinopathy	26 (18.6)	98 (16.3)	10 (14.9)	.764
Nephropathy	19 (13.6)	81 (13.5)	6 (9.0)	.545
Coronary heart disease	23 (16.4)	75 (12.5)	11 (16.4)	.399
Myocardial infarction	12 (8.6)	42 (7.0)	7 (10.5)	.562
Stroke	9 (6.4)	14 (2.3)	4 (6.0)	.027
Peripheral artery occlusive disease	15 (10.7)	39 (6.5)	7 (10.5)	.169
Diabetic foot syndrome	12 (8.6)	19 (3.2)	3 (4.5)	.018
Education attainment level - no. (%)				
Lower secondary education	86 (61.4)	243 (40.6)	21 (31.3)	<.001
Intermediate secondary education	19 (13.6)	127 (21.2)	14 (20.9)	
Upper secondary education	10 (7.1)	41 (6.8)	7 (10.5)	
Tertiary education	15 (10.7)	165 (27.5)	25 (37.3)	
Frequency of ophthalmologist consultations - no. per year (%)				
≥ 3×	23 (16.4)	55 (9.2)	5 (7.5)	.036
2×	36 (25.7)	131 (21.9)	12 (17.9)	
1×	62 (44.3)	273 (45.6)	34 (50.7)	
< 1×	16 (11.4)	125 (20.9)	14 (20.9)	

Regarding the preferred decision-making, there were no significant gender-specific differences.

A significant lower level of education was found in the supporters of ODM (*p* < 0.001). The percentage of those with lower secondary education was 66.2%, in contrast to 42.2% in the SDM group and only 31.3% in the PDM group. Accordingly, subjects with a higher level of education wished significantly more often to determine the preferred treatment alone (*p* < 0.001). Thus, the tertiary education level was 37.3% in the PDM group, 28.7% in the SDM group and 11.5% in the ODM group. With growing education level, increasingly the patients wanted to determine the therapy decision alone or together with the ophthalmologist.

Although there was a significant difference between the mean duration time in type 2 (23.6 years) and 1 diabetes (12.6 years), the decision-making process was not associated with duration of diabetes. Differences in the decision-making process between type 1 and 2 diabetes were related to their dependence on age and education.

There were no significant differences between the groups regarding glycated hemoglobin levels. Patients with DR did not let the ophthalmologist decide more frequently than those without. In the multivariate logistic regression analysis decision-making was not associated with diabetic comorbidities like retinopathy, nephropathy, neuropathy and coronary heart disease.

Some 27.9% of patients with low scores of DR knowledge within the interval zero to three wanted to delegate the treatment decision to the ophthalmologist (*p* = 0.008). In contrast, 75.7% of patients with higher scores for true answers wanted SDM and 15.1% ODM. Thus, the informed patient showed a higher propensity for SDM (Additional file 2).

A large majority of 82.6% memorized their HbA_1_c level. In the small group without knowledge of the HbA_1_c level, (27.7%) preferred ODM and (64.2%) SDM (*p* = 0.001). Patients who did not know their HbA_1_c level were more likely to transfer the decision-making competence to their treating ophthalmologist.

Displaying the percentage of the decision-making groups as a function of the frequency of ophthalmologist consultations per year the ODM group showed an increasing fraction from 10.3% to 27.3% with rising frequency rates from less than once per year to more than three times per year (*p* = 0.036). The more the patients consulted the ophthalmologist, the more they wanted the ophthalmologist to decide, which was coincident with decreasing values of the SDM option.

**Fig. 2:**
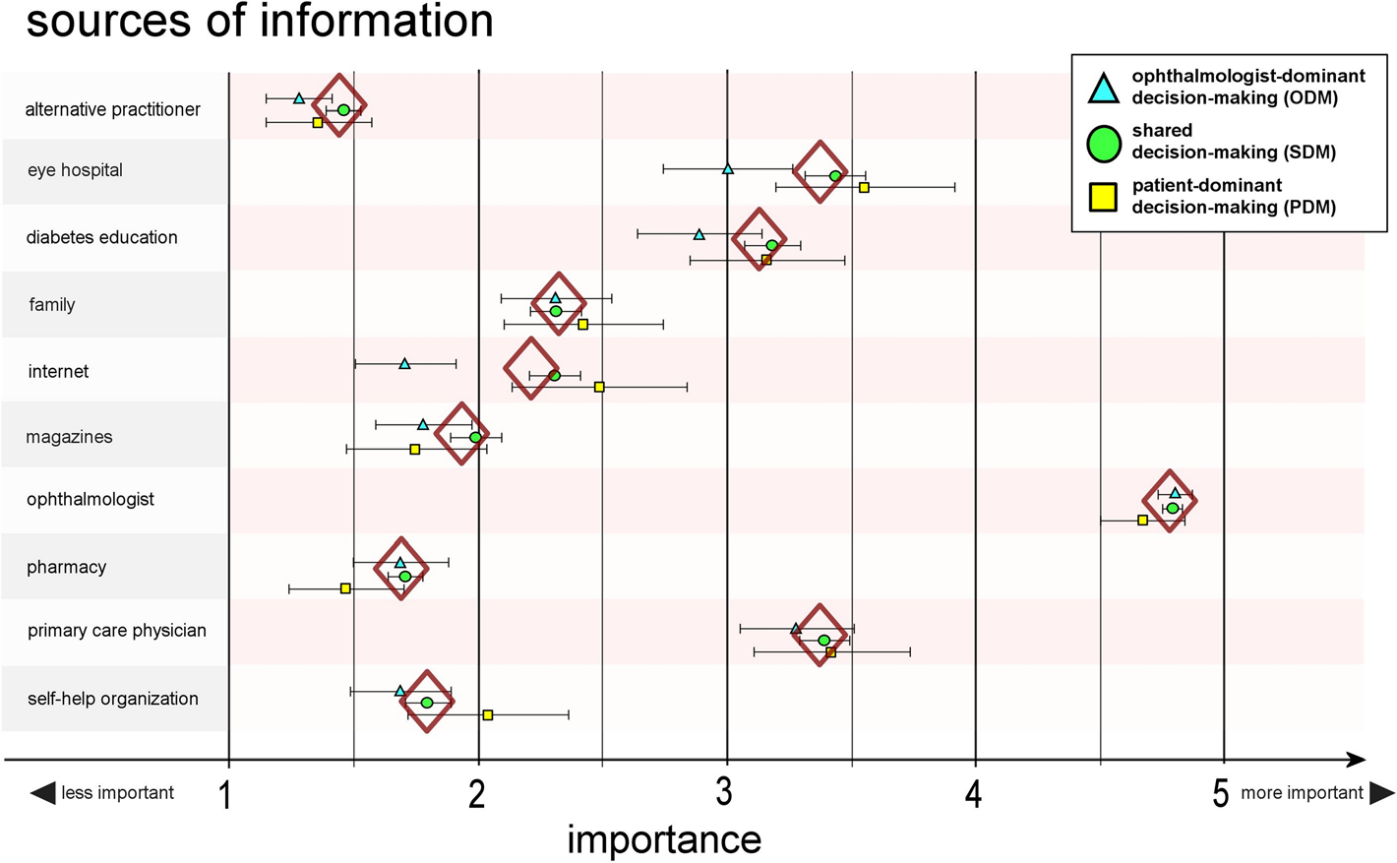
Members of the decision-making groups rated the importance attaching to the different sources of information. The whiskers extend to the 95% confidence intervals of the score values within the decision-making groups. The diamond is indicating the mean score value of all persons interviewed

For all groups the most important information source (scale 1–5) before treatment of the retina was the ophthalmologist with a mean value of 4.8 [95% CI, 4.75–4.81] followed by the eye hospital with a mean value of 3.4 [3.26–3.47], the general practitioner with a mean value of 3.4 [3.28–3.46] and diabetes education with a mean value of 3.1 [3.02–3.21]. Family, internet, journals, self-help group, pharmacist and alternative practitioner were all less important. However, the ODM group was less likely to prefer the internet and eye hospital as information sources relative to the SDM and PDM group (Fig. 2).

Multivariate logistic regression analysis was used to identify independent predictors of the decision-making preferences as shown in Table 2. The odds ratios are first reported for the ODM group and second for the PDM group both times with the SDM group as reference category.

**Table 2:** Predictors of the decision-making groups (Multivariate logistic regression analysis: ^a^The information sources self-help group and journals were not significant predictors of the ODM group. ^b^Age, education attainment and frequency of consultations were not significant predictors of the PDM group)

ODM vs SDM group^a^		
Factor	Odds ratio [95% CI]	*p* value
Age [yr]	1.02 [1.00–1.04]	.013
Education attainment [per level]	1.39 [1.15–1.69]	.001
Frequency of consultations	1.31 [1.04–1.65]	.022
Knowledge score of diabetic retinopathy and HbA_1_c level	0.90 [0.81–0.99]	.037
PDM vs SDM group^b^		
Factor	Odds ratio [95% CI]	*p* value
Education attainment [per level]	0.80 [0.66–0.99]	.036
Information sources		
self-help group	1.34 [1.06–1.70]	.015
Journals	0.70 [0.53–0.93]	.013

**Table 3:** Inter-group comparison of the different decision-making preference

	ODM (*n* = 119) vs SDM (*n* = 543) group		PDM (*n* = 62) vs SDM (*n* = 543) group	
Factor	OR [95% CI]	*p* value	OR [95% CI]	*p* value
Age [yr]	1.02 [1.00–1.04]	.013	1.00 [0.99–1.02]	.561
Education attainment [per level]	0.72 [0.59–0.87]	.001	1.24 [1.02–1.52]	.036
Frequency of consultations	1.31 [1.04–1.65]	.022	0.92 [0.66–1.27]	.603
Knowledge of diabetic retinopathy and HbA_1_c level [index]	0.90 [0.81–0.99]	.037	1.01 [0.88–1.17]	.867
Information sources				
self-help group	1.01 [0.82–1.23]	.957	1.34 [1.06–1.70]	.015
journals	0.90 [0.74–1.09]	.277	0.70 [0.53–0.93]	.013

Comparison of the decision-making process between two patients of 10 years age difference (delta = 10 yrs) showed that the older patient’s probability wishing the ophthalmologist to decide was 1.2 times higher as that of wanting SDM [OR = 1.02^(age-difference)^]. Comparison of lower secondary education versus tertiary education showed the OR of 2.7 for ODM [OR = 1.4^(level-difference)^]. Comparison of frequency of consultations demonstrated an OR of 2.2 for attendance of more than three times to less than one time per year [OR = 1.3^(frequency-difference)^]. Patients with a basic knowledge in DR who memorized their HbA_1_c level preferred SDM. The OR was 1.7 in the case of a five point score advantage (Δscore points = 5) within the knowledge score [OR = (1/0.9)^(number of correct answers)^].

Comparing the PDM with the SDM group the OR for tertiary education versus lower secondary education was 2.0 [OR = (1/0.8)^(level-difference)^] (Table 3). Patients who wanted to decide for themselves showed a higher propensity for self-help groups and less propensity for reading journals and newspapers (Fig. 2).

## Discussion

Except for the conservative treatment of glaucoma, the concept of decision-making and its consequences on adherence have not been evaluated for ophthalmology yet. In our survey of persons with diabetes, the overwhelming majority (74.3%) preferred SDM between ophthalmologist and patient, 17.4% patients wanted a passive role and only 8.3% preferred a patient-dominant treatment decision.

When assessing the results of the diabetes cohort, it is important to compare the results with other studies, reporting representative samples of the population in different countries. A large US population-based survey exploring the decision-making style in the general population showed 9% of respondents favored a passive style, 62% favored SDM and 28% preferred to make clinical decisions by themselves [17]. An annual representative sample of Germany between 2001 and 2012 showed between 14 and 22% for decision-making by the physician, between 52% and 58% for SDM, and an interval of 19% to 25% for the autonomous type of patients [23, 24]. Comparing these studies, based on the general population, with our study we found a lower percentage of patients preferred PDM. This might be because diagnosis and treatment have become a high-tech area with very specialized knowledge. Imaging techniques such as optical coherence tomography (OCT) have enhanced the early detection of macular edema in DR. Because of the increasingly specialized decisions diabetic patients have to face, they may tend to decide less autonomously leading to a smaller rate of PDM and more frequently wish to share the decision-making.

The diagnosis of a threatening disease may have an influence on the decision-making process. Degner and colleagues showed that 44% of women with breast cancer wanted to be involved in SDM with their physician, 34% preferred to delegate the responsibility to their physician and 22% wanted to choose the therapy alone [25]. Furthermore, studies of different disease populations showed a general tendency of patients to prefer a more passive role if they had diseases which could cause a severe deterioration of their health condition [26, 27].

Although the majority of the patients wanted SDM, patients’ preferences for involvement in decision-making showed variations because of the impact of sociodemographic variables, diabetes characteristics, frequency of ophthalmologist consultations and knowledge about DR and other diabetic ocular complications. In older patients with low educational attainment, the probability of opting for the more passive ODM is increasing. This result is consistent with previous research and has been found for different diseases [28–31].

In particular, the proportion of patients with lower secondary educational attainment among the 71–90 age group wanting ODM remained a minority, but increased from 17.4% (average) to 28.3% in this group. However, the majority of 63.7% still preferred the shared decision approach. This suggests that special attention should be paid to the fact that approximately a quarter of patients in this group even wanted to delegate the decision-making process to the ophthalmologist.

Little is known as yet about the frequency of physician consultations and the possible impacts on the decision-making process. We assumed that frequent consultations with the ophthalmologist would improve and deepen the doctor-patient-relationship with a better outcome in terms of information exchange about knowledge and treatment of DR. Indeed, we found a positive correlation (*p* < 0.01) between the patients’ knowledge score about DR and the frequency of their ophthalmologist consultations. Consequently, we would have expected a trend towards higher levels of SDM, but surprisingly, with increasing frequency of ophthalmologist consultations there was a small but growing proportion of those who prefer to rely on the ophthalmologist for medical decisions. Similar observations have been made indicating a more passive role in the course of patient career [23]. The slight increase of the ODM role with more frequent visits to the doctor could demonstrate the need of further improving patient-centered care including SDM. There are still gaps between patients’ desire for involvement in decision-making and their experience in reality [17, 18, 32, 33].

Knowledge of the patient’s HbA_1_c level by heart and higher levels of DR knowledge were factors increasing the propensity for SDM. Patients with a lack of basic knowledge in questions such as lower risk of retinopathy by improved metabolic control, diabetes-related complications in the retina, lack of early visual symptoms, and regular eye examinations tended to delegate the decision-making process to the ophthalmologist. Reasons for the reduced basic knowledge could be that patients only comprehend approximately 50% of the physician’s information, especially patients with low functional health literacy [34, 35]. Health literacy was defined as “the degree to which individuals have the capacity to obtain, process, and understand basic health information and services needed to make appropriate health decisions” [36, 37]. Low health literacy was associated with worse management of diabetes and higher rates of retinopathy among primary care patients with type 2 diabetes [38].

We see a certain need for further action mainly to ameliorate patient educational programs particularly concerning the implementation of ophthalmologic education.

In addition, ophthalmologist offering various options for targeted individual therapy should be aware of the patients’ perspective. The ophthalmologist could encourage active and self-determined setting of individualized treatment targets.

### Limitations

Data of a cross-sectional data is descriptive. Thus, causality cannot be derived. One limitation of our study was the recruitment in specialized diabetes care centers. Not only might those patients have more severe diabetes, but they might also enjoy better care than patients attending a general practitioner or no physician at all. The preferences of the decision-making process were not assessed during DR treatment, but the real as well as the virtual (as described in the questionnaire) can cause artefacts. Neither the presence of DR nor a performed treatment of the retina in the past were significant impact factors which have influenced the decision-making process of the 806 patients with diabetes, thus we would not expect important differences between the real and the hypothetical scenario. The cohort consisted of DR patients as well as future candidates at risk of DR.

As with other interview-based studies, there may be selection bias. However, the rejection rate was quite low and it is unlikely that the subjects were influenced to any large degree by the setting of practice rooms. Less than 3% of individuals declined to participate and this group did not differ from those enrolled with regard to demographic characteristics. Oral in-clinic interviews have the potential to introduce bias compared with other methods (e.g., telephone interviews, mail-in questionnaires) if patients feel pressured to provide socially acceptable answers or are concerned that their responses may be disclosed to their providers despite assurances by the investigators. Thus, the study may underestimate the percentage of subjects who want to take decisions by themselves. We attempted to minimize this bias by assuring patients of the anonymity of their responses and conducting their interviews in private.

## Conclusions

The concept of SDM before treatment of DR was favored by three quarters of our patients. A quarter of older patients with low educational attainment wanted to delegate the decision-making process to the ophthalmologist. A higher basic knowledge about DR increased patients’ propensity for SDM combined with a lower level of patients’ passive role in decision-making [39, 40]. Thus, amelioration of diabetic education programs is desirable, particularly concerning the implementation of ophthalmologic education.

Regardless of the decision-making process nearly all patients wanted medical and scientific information from the ophthalmologist before treatment of the retina and compared with other information sources the ophthalmologist was with great lead the most important one. The wishes and requirements of patients have to be considered for a patient-centered medicine [18].

## Additional files

Additional file 1 **Fig. S1.** Age distribution of the decision-making groups: The percentage of the ODM group is increasing with rising age, coincidently with decreasing values for the SDM group. (TIFF 65 kb)

Additional file 2: Questions of the Eye-Q questionnaire (NEI) and the phrases of the Control Preferences Scale (CPS). (DOC 28 kb)

## Abbreviations

DR: Diabetic retinopathy
HbA1_c_: Glycated hemoglobin
ODM: Ophthalmologist-dominant decision-making
PDM: Patient-dominant decision-making
SDM: Shared decision-making
