# Supplementary figures and images for: Patients’ preferences for involvement in the decision-making process for treating diabetic retinopathy
Source: BMC Ophthalmol. 2017 Aug 9;17:139. doi: 10.1186/s12886-017-0526-z (PMC5551005; doi:10.1186/s12886-017-0526-z)

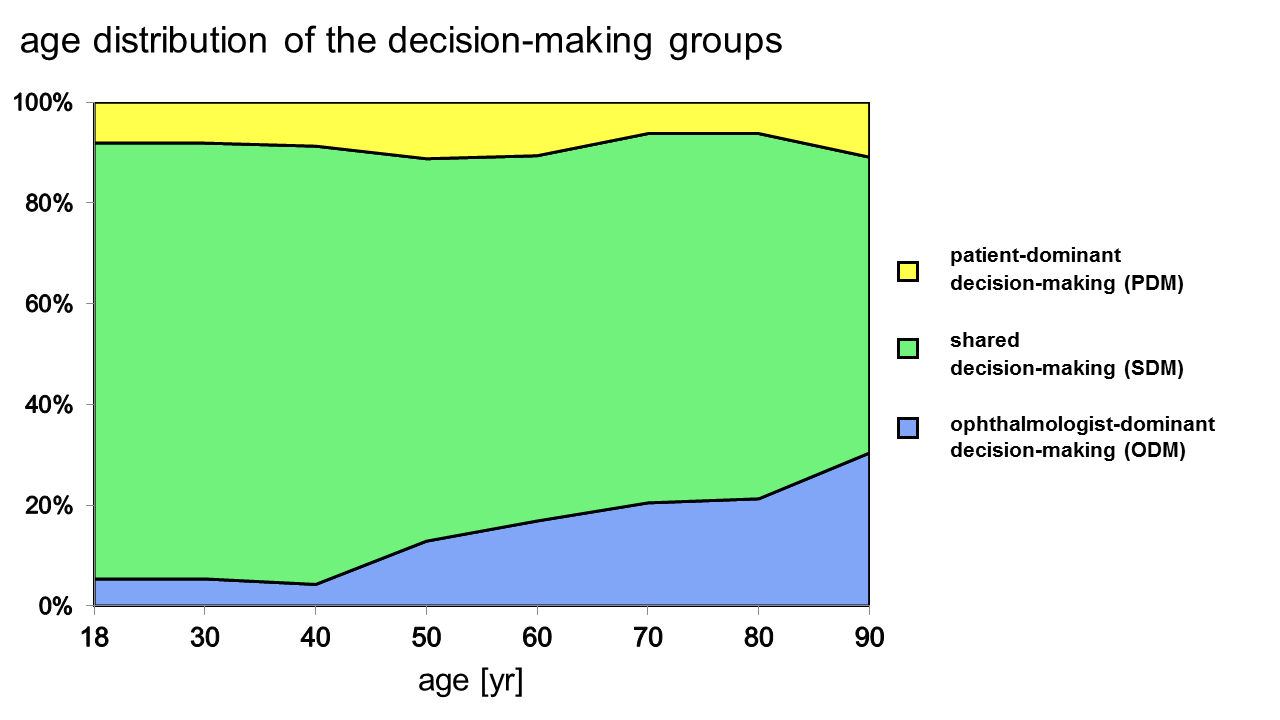

Supplement: Supplementary file 1 — Fig. S1. Age distribution of the decision-making groups: The percentage of the ODM group is increasing with rising age, coincidently with decreasing values for the SDM group. (TIFF 65 kb) [file 12886_2017_526_MOESM1_ESM.tif]
