# Supplementary material for: Patients’ preferences for involvement in the decision-making process for treating diabetic retinopathy
Source: BMC Ophthalmol. 2017 Aug 9;17:139. doi: 10.1186/s12886-017-0526-z (PMC5551005; doi:10.1186/s12886-017-0526-z)
Supplement: Supplementary file 2 — Questions of the Eye-Q questionnaire (NEI) and the phrases of the Control Preferences Scale (CPS). (DOC 28 kb) [file 12886_2017_526_MOESM2_ESM.doc]

**Eye – Q questionnaire (National Eye Institute)**

Please answer the following questions:

⬜ True ⬜ False ⬜ Not sure

- People with diabetes are more likely than people without diabetes to develop certain eye diseases.
- Diabetic eye disease usually has early warning signs.
- People with diabetes should have yearly eye examinations.
- People with diabetes are at low risk of developing glaucoma.
- Laser surgery can be used to halt the progression of diabetic retinopathy.
- Diabetic retinopathy is caused by changes in the blood vessels in the eye.
- People with diabetes are at low risk for developing glaucoma.
- Laser surgery can be used to halt the progression of diabetic retinopathy.
- Cataracts are common among people with diabetes.
- People who have good control of their diabetes are not at high risk for diabetic eye disease.
- The risk of diabetic eye disease can be reduced.

**Subjective Knowledge assessment**

“How well informed do you feel on eye diseases caused by diabetes?”

- "not informed at all”
- “less well informed”
- “mediocre”
- “well informed”
- “very well informed”

**Importance of Information source**

“Which source of information is important for you, when you weigh the advantages and disadvantages of a planned treatment of your eyes? Please rate the importance”

- "unimportant”
- “less important”
- “average”
- “more important”
- “very important”

**Control Preferences Scale (CPS) after Degner et al. 1997**

“Please imagine the following situation: The eye doctor discovers some damage to your retina caused by diabetes. There are several treatment options.

When deciding on what treatment is best for your health problems, what is your preferred role in treatment decision-making?”

- “I prefer to make the decision about which treatment I will receive”
- “I prefer to make the final decision about my treatment after seriously considering my doctor’s opinion”
- “I prefer that my doctor and I share the responsibility for deciding which treatment is best for me”
- “I prefer that my doctor make the final decision about which treatment will be used, but seriously considers my opinion”
- “I prefer to leave all decisions regarding treatment to my doctor”
